# Effects of Deacetylase Inhibition on the Activation of the Antioxidant Response and Aerobic Metabolism in Cellular Models of Fanconi Anemia

**DOI:** 10.3390/antiox12051100

**Published:** 2023-05-15

**Authors:** Nadia Bertola, Stefano Regis, Silvia Bruno, Andrea Nicola Mazzarello, Martina Serra, Michela Lupia, Federica Sabatini, Fabio Corsolini, Silvia Ravera, Enrico Cappelli

**Affiliations:** 1 Department of Experimental Medicine, University of Genoa, Via De Toni 14, 16132 Genova, Italy; 2 Laboratory of Clinical and Experimental Immunology, IRCCS Istituto Giannina Gaslini, Via Gerolamo Gaslini 5, 16148 Genova, Italy; 3 Haematology Unit, IRCCS Istituto Giannina Gaslini, Via Gerolamo Gaslini 5, 16148 Genova, Italy; 4 Stem Cell Laboratory and Cell Therapy Center, IRCCS Istituto Giannina Gaslini, Via Gerolamo Gaslini 5, 16148 Genova, Italy; 5 Centro di Diagnostica Genetica e Biochimica delle Malattie Metaboliche, IRCCS Istituto Giannina Gaslini, Via Gerolamo Gaslini 5, 16148 Genova, Italy

**Keywords:** catalase, glutathione reductase, oxidative phosphorylation, lipid peroxidation, aldehyde dehydrogenase, energy metabolism, antioxidant defenses, histone deacetylase inhibitors

## Abstract

Fanconi anemia (FA) is a rare genetic disease characterized by a dysfunctional DNA repair and an oxidative stress accumulation due to defective mitochondrial energy metabolism, not counteracted by endogenous antioxidant defenses, which appear down-expressed compared to the control. Since the antioxidant response lack could depend on the hypoacetylation of genes coding for detoxifying enzymes, we treated lymphoblasts and fibroblasts mutated for the *FANC-A* gene with some histone deacetylase inhibitors (HDACi), namely, valproic acid (VPA), beta-hydroxybutyrate (OHB), and EX527 (a Sirt1 inhibitor), under basal conditions and after hydrogen peroxide addition. The results show that VPA increased catalase and glutathione reductase expression and activity, corrected the metabolic defect, lowered lipid peroxidation, restored the mitochondrial fusion and fission balance, and improved mitomycin survival. In contrast, OHB, despite a slight increase in antioxidant enzyme expressions, exacerbated the metabolic defect, increasing oxidative stress production, probably because it also acts as an oxidative phosphorylation metabolite, while EX527 showed no effect. In conclusion, the data suggest that VPA could be a promising drug to modulate the gene expression in FA cells, confirming that the antioxidant response modulation plays a pivotal in FA pathogenesis as it acts on both oxidative stress levels and the mitochondrial metabolism and dynamics quality.

## 1. Introduction

Fanconi anemia is a genomic instability syndrome mainly characterized by bone marrow failure [1,2] and cancer predisposition [3,4,5] due to an impairment in DNA interstrand crosslink (ICLs) repair [6,7]. In addition, FA cells show dysfunctional oxidative phosphorylation (OxPhos) [8,9,10] caused by an altered electron transport between respiratory complexes I and III [11,12,13]. Because of the aerobic metabolism impairment, FA cells show a metabolic switch toward lactate fermentation [14] and acetyl-CoA accumulation that causes an increase in fatty acid synthesis and lipid droplet accumulation [15]. The dysfunctional mitochondrial metabolism in FA cells is also associated with high oxidative stress production [9,16,17], which represents one of the causes of ICLs [18]. In addition, FA cells appear unable to counteract the oxidative stress increment since the expression and the activity of endogenous antioxidant defenses, such as catalase, glutathione reductase, glutathione peroxidase, and aldehyde dehydrogenase, is lower than in healthy cells and does not increase after oxidative stress insult [13,17].

Gene expression is regulated by different chromatin modifications [19], including acetylation, which plays a pivotal role in transcriptional regulation as it modulates the chromatin accessibility of transcriptional and DNA repair proteins [20]. The acetylation status depends on the balance between the deacetylase (HDAC) and acetyltransferase (HAT) enzymatic activity [21]. While histone acetylation leads to increased gene expression, deacetylation has the opposite effect. Moreover, acetylation represents a fundamental post-translational modification that determines different proteins’ functions or localization [22]. So far, there are 18 HDAC enzymes subdivided into four classes [23,24]. Classes I, II, and IV are zinc dependent, and III is NAD^+^ dependent [23]. Histone deacetylase inhibitors (HDACi) modify gene expression at many levels [25] and are used in several diseases, such as chronic inflammatory disorders [26,27,28,29,30], cancers [31,32,33,34], and neurodegenerative diseases [35,36].

The role of chromatin modifications in FA cells remains slightly investigated. Renaud et al. show that chromatin in FA cells is hypoacetylated, allowing the localization of 53BP1, a mammalian non-homologous DNA end-joining (NHEJ) protein, on DNA double-strand breaks (DBS) [37]. Treatment with an HDACi counteracts the cytotoxic effect of NHEJ, preventing the localization of 53BP1 protein on DNA DSB and leaving free access to the homologous recombination protein [38,39].

Thus, this work investigated the HDACi effects on antioxidant response and energy metabolism in lymphoblasts and fibroblasts carrying the mutation for the *FANC-A* gene. In particular, FA lymphoblasts and fibroblasts were treated with three different HDACi: valproic acids (VPA), beta-hydroxybutyrate (OHB), and EX527.

Our results show that VPA and, to a lesser extent, OHB induce the expression and activity of catalase and glutathione reductase, enzymes involved in the antioxidant response in FA cells. However, only VPA improves mitochondrial metabolism, reducing oxidative stress and MMC sensibility. Conversely, OHB causes a loss of OxPhos efficiency and increases the accumulation of lipid droplets and malondialdehyde, whereas EX527 does not affect energy metabolism and antioxidant defense activity.

## 2. Materials and Methods

### 2.1. Materials

All chemical compounds were of the highest chemical grade and were purchased from Sigma-Aldrich (St. Louis, MO, USA). 

### 2.2. HDACi Treatment

To inhibit histone deacetylases (HDAC), three different molecules were employed: valproic acid (VPA), beta-hydroxybutyrate (OHB), and EX527. In detail, VPA is a drug used for seizures and bipolar disorder [40], which inhibits class I (HDAC1, 2, 3, and 8) and class IIa (HDAC4, 5, 7, and 9) [41], providing an anti-cancer effect [42]. The ketone body OHB is a specific class I HDACi (HDAC1 and HDAC2) that promotes the transcription of catalase and mitochondria super-oxide dismutase genes by increasing the expression of FoxO3A and MT2 genes, two oxidative stress resistance factors [43]. EX527 is a specific inhibitor of sirtuin 1 (SIRT1) and, to a lesser extent, sirtuin 6 (SIRT6) [44,45], two HDAC belonging to class III deacetylases regulating the response to stress and mitochondrial biogenesis [46].

In this manuscript, both lymphoblast and fibroblast cell lines were treated with 1 mM VPA [47], 10 mM OHB [43,48], or 100 nM EX257 [44]. Drugs’ effects on antioxidant defenses and aerobic metabolism were investigated 3 or 24 h after the treatment. Specifically, at the 3 h time point, the effects of short-term HDACi treatment on the expression and activity of antioxidant enzymes were evaluated; experiments after 24 h from the treatment were performed to assess whether the antioxidant defense restoration could promote and improve oxidative metabolism.

### 2.3. Cell Culture 

Six different *FANC-A* lymphoblast cell lines (Lympho FA) and three different *FANC-A* primary fibroblast cell lines (Fibro FA) derived from six patients who carried out different mutations of the *FANC-A* gene were obtained from the ‘‘Cell Line and DNA Biobank from Patients affected by Genetic Diseases’’ (G. Gaslini Institute)—Telethon Genetic Biobank Network (Project No. GTB07001). As controls, isogenic FA-corr cell lines generated by the same *FANC-A* lymphoblast and fibroblast cell lines corrected with S11FAIN retrovirus (Lympho FA-corr and Fibro FA-corr) were employed [12,49]. In addition, lymphoblast cell lines and primary fibroblasts isolated from healthy donors were also employed to demonstrate that isogenic corrected cells represent a proper control. The data reported in Figures S1 and S2, respectively, showed no difference between healthy and FA-corr cells in the biochemical readouts used in this manuscript.

For lymphoblast cell lines, RPMI-1640 medium (#21875091, GIBCO, Billing, MT, USA) containing 10% FBS (#ECS0120L, Euroclone, Milano, Italy), 100 U/mL penicillin, and 100 μg/mL streptomycin (#ECB3001D, Euroclone, Milano, Italy) was used, and the cells were grown at 37 °C with a 5% CO_2_ [12]. Primary fibroblasts were cultured as a monolayer in DMEM high glucose with glutamax ^®^ (#61965026, GIBCO, Billing, MT, USA), containing 10% fetal bovine serum (FBS; #ECS0120L, Euroclone, Milano, Italy), 100 U/mL penicillin, and 100 μg/mL streptomycin (#ECB3001D, Euroclone, Milano, Italy) at 37 °C with a 5% CO_2_. 

When necessary, 0.5 mM hydrogen peroxide (H_2_O_2_) was added to induce oxidative damage.

### 2.4. Gene Expression Evaluation 

RNA was extracted from lymphoblast pellets using the RNeasy mini kit (Qiagen, Hilden, Germany). To evaluate the expression of catalase (*CAT*) and glutathione reductase (*GR*) mRNAs, 100 ng of RNA was reverse-transcribed using the SuperScript VILO IV cDNA Synthesis Kit (Invitrogen, Waltham, MA, USA). Real-time PCR was performed on the cDNA using the primers-probe mix contained in the specific TaqMan Gene Expression Assays (Applied Biosystems, Waltham, MA, USA). Gene expression was normalized to the *GAPDH* expression. Experiments were performed in triplicate.

### 2.5. Cell Homogenate Preparation and Treatment

After two washes in PBS, primary fibroblasts and lymphoblasts pellets were resuspended in PBS supplied with protease inhibitors (P2714, Sigma-Aldrich, St. Louis, MO, USA) and sonicated (Microson XL Model DU-2000, Misonix Inc., Farmingdale, NY, USA) twice for 10 s, with a 30 s break and in ice to prevent heating. Total protein content was assayed by the Bradford method [50]. 

### 2.6. Antioxidant Enzyme Activity Evaluation

Catalase (CAT), glutathione reductase (GR), and aldehyde dehydrogenase (ALDH) were assayed as markers of cellular antioxidant defenses. For each assay, 50 μg of total proteins was used.

CAT activity was assayed spectrophotometrically following the H_2_O_2_ decomposition at 240 nm. The assay mix contained 50 mM phosphate buffer (pH 7.0) and 5 mM H_2_O_2_ [51]. 

GR activity was assayed spectrophotometrically at 340 nm, following the NADPH oxidation. The assay medium contained 100 mM Tris-HCl (pH 7.4), 1 mM EDTA, 5 mM GSSG, and 0.2 mM NADPH [52].

ALDH activity was measured spectrophotometrically at 340 nm following the NAD^+^ oxidation. The assay mix contained 100 mM sodium pyrophosphate (#221368 Sigma-Aldrich, St. Louis, MO, USA) at pH 9, 10 mM NAD^+^ (N0632 Sigma-Aldrich, St. Louis, MO, USA), and 10 mM acetaldehyde (#402788 Sigma-Aldrich, St. Louis, MO, USA) [53].

### 2.7. Lipid Peroxidation Evaluation

Lipid peroxidation damage was evaluated spectrophotometrically at 532 nm by the malondialdehyde (MDA) concentration assay, using the thiobarbituric acid reactive substance (TBARS) assay. For each sample, 50 μg of total protein was dissolved in 300 μL of Milli-Q water and 600 μL of TBARS solution [54].

### 2.8. Lipid Content Evaluation

The lipid content was evaluated spectrophotometrically at 535 nm by the Sulfo-Phospho-Vanillin assay, according to [13]. A mix of triglycerides (#17810, Sigma-Aldrich, St. Louis, MO, USA) was employed for the standard curve.

### 2.9. Evaluation of Electron Transport between Complexes I and III 

The electron transfer between complex I and complex III was assayed spectrophotometrically at 550 nm following the reduction of cytochrome c on 50 μg of total protein. The reaction mix contained 100 mM Tris-HCl (pH 7.4), 0.03% of oxidized cytochrome c (#C2867, Sigma-Aldrich, St. Louis, MO, USA), and 0.7 mM NADH [55]. 

### 2.10. ATP and AMP Intracellular Content Evaluation

For each assay, 50 μg of total protein was used. 

ATP was assayed spectrophotometrically following NADP reduction at 340 nm. Assay medium contained 100 mM Tris-HCl (pH 8.0), 0.2 mM NADP, 5 mM MgCl_2_, and 50 mM glucose. Samples were analyzed before and after the addition of 3 μg of purified hexokinase plus glucose-6-phosphate dehydrogenase [56]. 

AMP was assayed spectrophotometrically following NADH oxidation at 340 nm. Reaction medium contained 100 mM Tris-HCl (pH 8.0), 5 mM MgCl_2_, 0.2 mM ATP, 10 mM phosphoenolpyruvate, 0.15 mM NADH, 10 IU adenylate kinase, 25 IU pyruvate kinase, and 15 IU lactate dehydrogenase [56]. 

The ratio between the intracellular concentrations of ATP and AMP (ATP/AMP) represents a marker of cellular energy status.

### 2.11. Oxidative Phosphorylation Evaluation 

Oxidative phosphorylation (OxPhos) metabolism was evaluated as oxygen consumption rate (OCR) and ATP synthesis through FoF1 ATP-synthase. For each assay, 105 cells, permeabilized with 0.03 mg/mL digitonin for 1 min, were employed. In both cases, 10 mM pyruvate plus 5 mM malate (#P4562 and #M8304, respectively, Sigma-Aldrich, St. Louis, MO, USA) or 20 mM succinate (#S7501, Sigma-Aldrich, St. Louis, MO, USA) was used as a respiratory substrate to stimulate the respiratory pathway led by Complex I or by Complex II, respectively.

OCR was measured with an amperometric electrode (Unisense Microrespiration, Unisense A/S, Aarhus, Denmark) [14,57]. 

ATP synthesis was monitored with a luminometer (GloMax ^®^ 20/20 Luminometer, Promega Italia, Milano, Italy) by the luciferin/luciferase chemiluminescent method (luciferin/luciferase ATP bioluminescence assay kit CLS II, Roche, Basilea, Switzerland) for 2 min every 30 s. ATP standard solutions in a concentration range between 10^−8^ and 10^−5^ M were used for the calibration curve [14,57].

### 2.12. Mitochondrial Energy Metabolism Efficiency Calculation 

To evaluate the OxPhos efficiency, the ratio between the aerobic synthesized ATP and the consumed oxygen (P/O) was calculated. Efficient mitochondria displayed a P/O value of around 2.5 or 1.5 when stimulated with pyruvate and malate or succinate, respectively. A P/O ratio lower than these values indicates that oxygen is not completely devoted to energy production but contributes to reactive oxygen species (ROS) formation [58]. 

### 2.13. Confocal Microscopy Analysis

Cells were cultured in chamber slides for 24 h in the absence or presence of the three HDACi. After fixation with 0.3% paraformaldehyde (#P6148, Sigma-Aldrich, St. Louis, MO, USA) and permeabilization with with 0.1% triton (#X100, Sigma-Aldrich, St. Louis, MO, USA), cells were incubated with anti-TOM20 antibody (#42406S, Cell Signaling Technology, Danvers, MA, USA) overnight at 4 °C. Afterward, cells were incubated for 1 h at 25 °C with the Alexa-546-conjugated anti-rabbit antiserum (#A11010, Invitrogen, USA) as a secondary antibody.

Immunofluorescence confocal laser scanner microscopy (CLSM) imaging was carried out using a laser scanning spectral confocal microscope TCS SP2 AOBS (Leica, Germany), equipped with Argon ion, He–Ne 543 nm, and He–Ne 633 nm lasers. Images were acquired through an HCX PL APO CS 40×/1.25 oil UV objective and processed with Leica. Images were acquired as single transcellular optical sections.

To evaluate the mitochondrial network shape, fibroblasts were scored depending on the morphology of most of their mitochondrial population as elongated or intermediated/short following the method described in [59].

### 2.14. Western Blot Analysis 

After denaturing electrophoresis (SDS-PAGE), nitrocellulose membrane was incubated with anti-MFN2 (#11925S, Cell Signaling Technology, Danvers, MA, USA), anti-DRP1 (#ab184247, Abcam, Cambridge, UK), anti-UCP2 (#sc-6525, Santa Cruz Biotechnology, Dallas, TX, USA), and anti-Actin (#sc-1616, Santa Cruz Biotechnology, Dallas, TX, USA). All primary antibodies were diluted in PBS plus 0.15% Tween (PBSt, Tween was from Roche, Basilea, Switzerland, #11332465001). Secondary antibodies (#A0168 and #SAB3700870, Sigma-Aldrich, St. Louis, MO, USA) were diluted 1:10,000 in PBSt. Bands were evaluated by a chemiluminescence system (Alliance 6.7 WL 20M, UVITEC., Cambridge, UK) using an enhanced chemiluminescence substrate (ECL, #1705061, BioRad, Hercules, CA, USA). Band intensity was evaluated by Alliance^TM^ Q9-Series software (UVITEC, Cambridge, UK). All the signals were normalized versus the actin signal. 

### 2.15. Mitomycin C Survival Assay

Lymphoblast cell line survival under mitomycin C (MMC) treatment (0, 1, 3, 10, and 33 nM) was tested as previously described in Bottega et al. [60].

### 2.16. Statistical Analysis

Data were analyzed by one-way ANOVA followed by Tukey’s multiple comparison test by Prism 8 Software. Data are expressed as mean ± standard deviation (SD) and are representative of 6 independent experiments. Errors with a probability of *p* < 0.05 were considered significant.

## 3. Results

### 3.1. VPA and OHB Treatment Enhanced Catalase and Glutathione Reductase Expression and Activity in Cells Mutated for the FANC-A Gene

**Figure 1:**
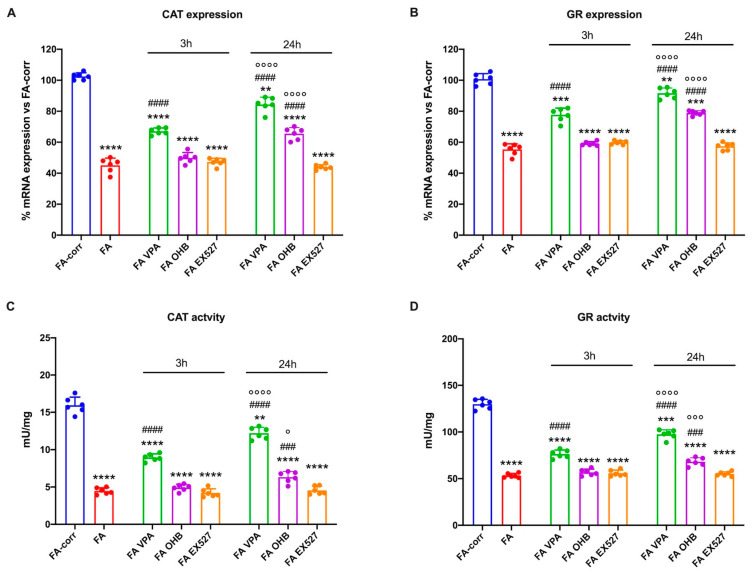
Modulation of catalase and glutathione reductase expression and activity in FA lymphoblasts treated with VPA, OHB, or EX527. (**A**) Catalase (CAT) gene expression; (**B**) glutathione reductase (GR) gene expression; (**C**) CAT activity; (**D**) GR activity. In each panel, the effects were evaluated in lymphoblasts after 3 and 24 h from VPA, OHB, or EX527 addition. Data are reported as mean ± SD, and each graph is representative of 6 independent experiments. Statistical significance was tested with a one-way ANOVA. **, ***, and **** represent a significant difference for *p* < 0.01, 0.001, or 0.0001, respectively, between FA and FA-corr cells used as control. ### and #### represent a significant difference for *p* < 0.001 or 0.0001, respectively, between untreated and treated FA cells. °, °°°, and °°°° represent a significant difference for *p* < 0.05, 0.001, or 0.0001, respectively, between the same treatment at 3 and 24 h.

Figure 1 shows that expression and activity of CAT (Panels A and C) and GR (Panels B and D), two enzymes involved in cellular antioxidant responses, were low in lymphoblasts mutated for the *FANC-A* gene with respect to the FA-corr cells, confirming the inability of FA cells to counteract the oxidative stress production by enhancing the antioxidant defenses [13].

Thus, to improve the expression and activity of enzymes involved in oxidative stress detoxification, FA cells were treated with VPA, OHB, or EX527, three histone deacetylase inhibitors [41,43,45]. The results show that, already after 3 h of treatment, VPA caused an enhancement in CAT and GR expression and activity in FA lymphoblasts, which was even more evident 24 h after the addition of the drug (Figure 1A,C). CAT and GR expression and activity also increased with OHB, but only after 24 h of treatment (Figure 1B,D). However, the drug-induced improvement did not reach the FA-corr cell level in both cases. By contrast, treatment with EX527 did not affect the expression and activity of the two enzymes (Figure 1). The same results on enzymatic activity were observed in primary fibroblasts (Figure S3).

### 3.2. VPA but Not OHB and EX527 Improved the lipid Profile and Mitochondrial Energy Metabolism in Cells Carrying the FANC-A Mutation

**Figure 2:**
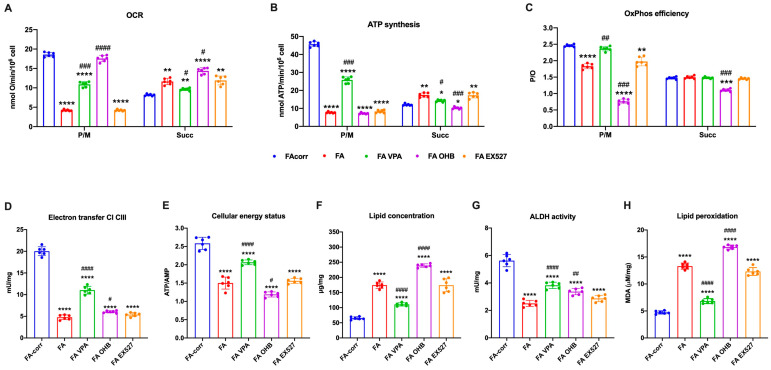
VPA, OHB, and EX527 effects on energy metabolism parameters and lipid peroxidation in FA lymphoblasts. (**A**) Oxygen Consumption Rate (OCR); (**B**) ATP synthesis through F_o_F_1_ ATP synthase; (**C**) P/O ratio as an OxPhos efficiency marker. For Panels A-C, pyruvate/malate (P/M) and succinate (Succ) were employed as respiratory substrates. (**D**) Electron transfer between complexes I and III; (**E**) ATP/AMP ratio as a cellular energy status marker; (**F**) cellular lipid concentration; (**G**) aldehyde dehydrogenase (ALDH) activity; (**H**) malondialdehyde (MDA) intracellular concentration as a lipid peroxidation marker. In each panel, the effects were evaluated in lymphoblasts after 24 h from the VPA, OHB, or EX527 addition. Data are reported as mean ± SD, and each graph is representative of 6 independent experiments. Statistical significance was tested opportunely with a one-way ANOVA. *, **, ***, and **** represent a significant difference for *p* < 0.05, 0.01, 0.001, or 0.0001, respectively, between FA and FA-corr cells used as control. #, ##, ###, and #### represent a significant difference for *p* < 0.05, 0.01, 0.001, or 0.0001, respectively, between untreated and treated FA cells.

The unfunctional antioxidant response exacerbated the metabolic alteration of FA cells, as a vicious circle was created between uncontrolled oxidative stress production and mitochondrial metabolic dysfunction [61,62]. Thus, several metabolic markers were evaluated after 24 h of treatment on lymphoblast cell lines (Figure 2) and primary fibroblasts (Figure S4) to investigate whether increased antioxidant enzyme activity also improved the energy metabolism defect.

The VPA addition enhanced OCR and ATP synthesis (Figure 2A,B and Figure S4A,B), although FA-corr cell values were not achieved. The aerobic metabolism amelioration improved the OxPhos efficiency, as suggested by the recovery of P/O ratio values in the presence of pyruvate/malate (Figure 2C and Figure S4C) thanks to the enhancement of the electron transfer between respiratory complexes I and III (Figure 2D and Figure S4D). The mitochondrial function recovery caused, in turn, an improvement of cellular energy status, as shown by the increase in the ATP/AMP ratio (Figure 2E and Figure S4E) and a reduction in lipid content (Figure 2F and Figure S4F), as better functioning OxPhos does not determine acetyl-CoA accumulation and its conversion in fatty acids. 

In addition, VPA increased the ALDH activity, an enzyme involved in aldehydes detoxification (Figure 2G and Figure S4G). The oxidative stress detoxification improvement and reduced intracellular lipid concentration could explain the decrease in lipid peroxidation (Figure 2H and Figure S4H).

By contrast, OHB treatment caused an increase in the uncoupling between oxygen consumption and energy production, as suggested by the OCR increment and the ATP synthesis decrease in the presence of both respiratory substrates (Figure 2A,B and Figure S4A,B), further reducing both P/O ratio values compared to untreated FA cells (Figure 2C and Figure S4C). Therefore, despite a slight electron transfer increment between complexes I and III (Figure 2D and Figure S4D), the cellular energy state did not improve (Figure 2E and Figure S4E), and the unfunctional OxPhos increased the lipid content (Figure 2F and Figure S4F). Furthermore, OHB did not affect the activity of ALDH (Figure 2G and Figure S4G), nor those of CAT and GR, causing an accumulation of lipid peroxidation (Figure 2H and Figure S4H), as the uncoupling of OxPhos is not counteracted by antioxidant defenses. 

No effects on the metabolic parameters were observed for EX527 treatment (Figure 2 and Figure S4).

**Figure 3:**
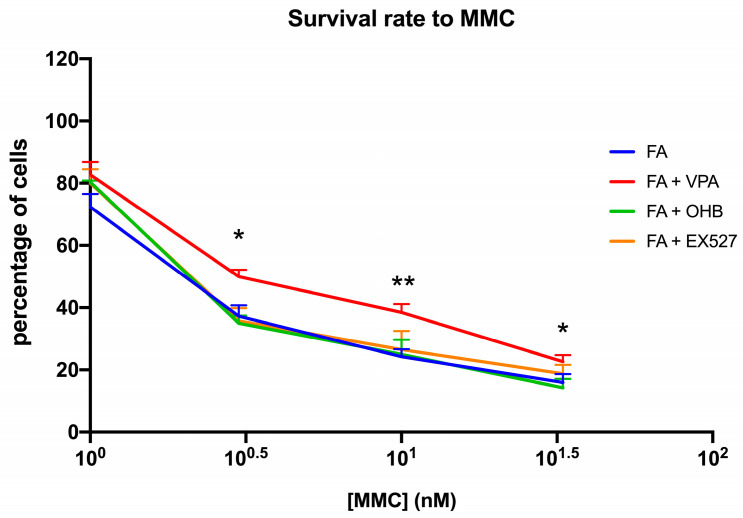
VPA, OHB, or EX527 treatment effect on mitomycin survival. The graph shows the mitomycin (MMC) survival rate of FA lymphoblasts treated with VPA, OHB, or EX527. Data are reported as mean ± SD, and each graph is representative of 6 independent experiments. Statistical significance was tested with a one-way ANOVA. * and ** represent a significant difference for *p* < 0.05 or 0.01, respectively, between FA and FA-corr cells used as control.

Interestingly, positive effects of VPA treatment on biochemical parameters were also seen in the ability of FA lymphoblasts to survive MMC (Figure 3).

### 3.3. VPA Treatment Improved the Balance between Fusion and Fission and Reduced the UCP2 Expression, Recovering the Mitochondrial Network Shape

**Figure 4:**
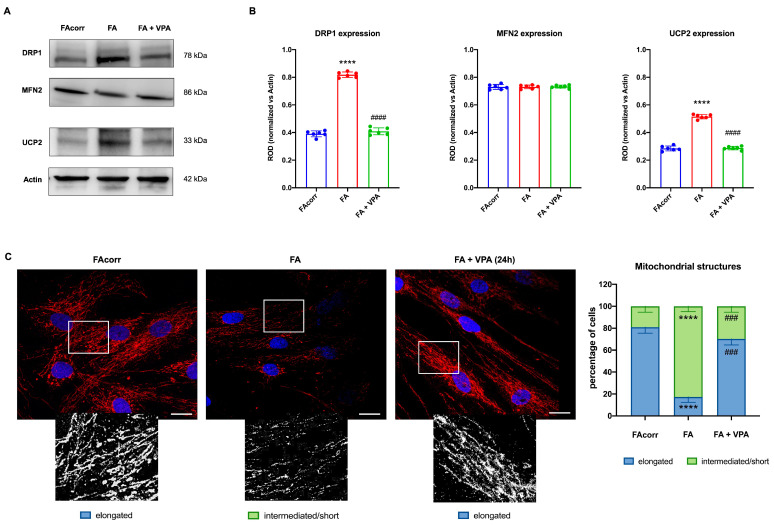
VPA modulation of mitochondrial dynamic and uncoupling protein 2 expression in FA primary fibroblasts. (**A**) WB signals and (**B**) relative densitometry of DRP1 (mitochondrial fission), MFN2 (mitochondrial fusion), uncoupling protein 2 (UCP2), and Actin (housekeeping protein used for signal normalization) in FA-corr and FA primary fibroblasts treated or not with VPA for 24 h. Each signal was normalized on the actin signal. (**C**) Confocal imaging of FA-corr and FA fibroblasts stained with antibody against TOM20 (red) and DAPI (blue) to show the mitochondrial reticulum and nuclei, respectively. White scale bars correspond to 10 μm. The higher magnification inserts, corresponding to the area enclosed by the white square, represent an example of mitochondrial network distribution. Data reported in the histogram on the right are expressed as mean ± SD. Each panel is representative of at least 6 independent experiments. Statistical significance was tested with a one-way ANOVA. **** represents a significant difference for *p* < 0.0001 between FA cells and FA-corr. ### and #### represent a significant difference for *p* < 0.001 or 0.0001, respectively, between untreated and VPA-treated FA fibroblasts.

FA cells are characterized by a disrupted mitochondrial network [63], in which organelles appear swollen with less defined cristae [12,63,64]. These structural alterations could be associated with unbalanced mitochondrial dynamics, as suggested by the WB analyses (Figure 4A,B). In particular, FA fibroblasts express a high level of DRP1, a protein involved in mitochondrial fission, compared to the control. Conversely, levels of MFN2, a protein involved in fusion, are similarly expressed in FA-corr and FA cells, causing an unbalanced in the mitochondrial network organization towards the fission as confirmed by the confocal images (Figure 4C). In addition, also the uncoupling protein 2 (UCP2) was found to be overexpressed in the FA fibroblast, justifying the low OxPhos efficiency (Figure 4A,B). In addition, Figure 4C shows that FA fibroblasts more often exhibited intermediated/short and less elongated mitochondria compared to FA-corr cells. Interestingly, treatment with VPA recovered DRP1 and UCP2 levels similar to the control cells (Figure 4A,B), restoring the elongated structures and, thus, the mitochondrial network shape organization (Figure 4C). 

### 3.4. FA Cells Were Unable to Counterbalance the Effects of a Pro-Oxidant Event except in the Presence of VPA

Considering that antioxidant defenses increase in response to an oxidative insult, the VPA, OHB, or EX527 effects were evaluated in FA-corr and FA-lymphoblastoid lines treated with hydrogen peroxide.

**Figure 5:**
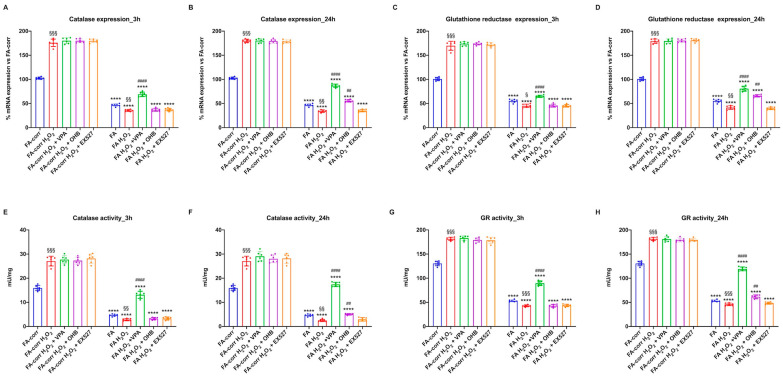
Modulation of catalase and glutathione reductase expression and activity in FA-corr and FA lymphoblasts treated with VPA, OHB, or EX527 after the hydrogen peroxide addition. In each graph, 0.5 mM hydrogen peroxide was added to induce oxidative insult. (**A**,**B**) Catalase (CAT) expression after 3 h and 24 h from VPA, OHB, or EX527 addition. (**C,D**) Glutathione reductase (GR) expression after 3 h and 24 h from VPA, OHB, or EX527 addition. (**E**,**F**) Catalase (CAT) activity after 3 h and 24 h from VPA, OHB, or EX527 addition. (**G**,**H**) Glutathione reductase (GR) activity after 3 h and 24 h from VPA, OHB, or EX527 addition. Data are reported as mean ± SD, and each graph is representative of 6 independent experiments. Statistical significance was tested with a one-way ANOVA. **** represents a significant difference for *p* < 0.0001 between FA and FA-corr cells in the same treatment condition. ## and #### represent a significant difference for *p* < 0.01 or 0.0001, respectively, between untreated and VPA-, OHB-, or EX537-treated samples. §, §§, and §§§ represent a significant difference for *p* < 0.05, 0.01, or 0.001, respectively, between H_2_O_2_-treated and untreated samples.

The results show that FA-corr lymphoblasts increased the expression and activity of CAT and GR already after 3 h of H_2_O_2_ treatment (Figure 5A,C,E,G), maintaining elevated defenses even 24 h after the oxidative damage (Figure 5B,D,F,H). Furthermore, the observed increase did not improve after the VPA, OHB, or EX527 addition, suggesting that control cells themselves trigger an adaptive response to oxidative stress and that HDCAi are not required to raise defenses.

In contrast, FA lymphoblasts, in the presence of H_2_O_2_, further decreased low CAT and GR expression and activity, probably due to the accumulation of oxidative damage. However, VPA, even under pro-oxidant conditions, induced CAT and GR expression and activity improvement after 3 h and 24 h of treatment. OHB also caused an enhancement in antioxidant defenses, but only after 24 h of treatment, whereas EX527 showed no effect. Similar results were obtained on primary fibroblasts (Figure S5).

**Figure 6:**
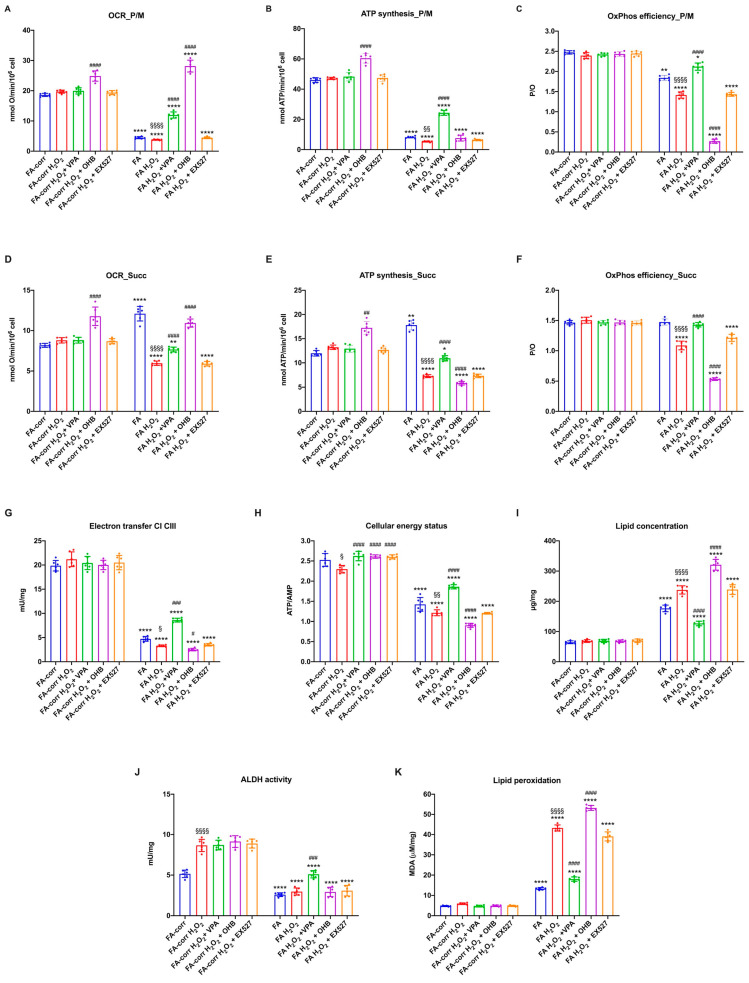
VPA, OHB, and EX527 effects on energy metabolism parameters and lipid peroxidation in FA-corr and FA lymphoblasts after hydrogen peroxide addition. In each graph, 0.5 mM hydrogen peroxide was added to induce oxidative damage. (**A**) Oxygen Consumption Rate (OCR) in the presence of pyruvate/malate (P/M). (**B**) ATP synthesis through F_o_F_1_ ATP synthase in the presence of P/M. (**C**) P/O ratio in the presence of P/M as an OxPhos efficiency marker. (**D**) Oxygen Consumption Rate (OCR) in the presence of succinate (Succ). (**E**) ATP synthesis through F_o_F_1_ ATP synthase in the presence of Succ. (**F**) P/O ratio in the presence of Succ as an OxPhos efficiency marker. (**G**) Electron transfer between complexes I and III. (**H**) ATP/AMP ratio as a cellular energy status marker. (**I**) Cellular lipid concentration. (**J**) Aldehyde dehydrogenase (ALDH) activity. (**K**) Malondialdehyde (MDA) intracellular concentration as a lipid peroxidation marker. In each panel, the effects were evaluated in lymphoblasts after 24 h from VPA, OHB, or EX527 addition. Data are reported as mean ± SD, and each graph is representative of 6 independent experiments. Statistical significance was tested opportunely with a one-way ANOVA or two-way ANOVA. *, **, and **** represent a significant difference for *p* < 0.05, 0.01, or 0.0001, respectively, between FA and FA-corr cells in the same treatment condition. #, ##, ###, and #### represent a significant difference for *p* < 0.05, 0.01, 0.001, or 0.0001, respectively, between untreated and VPA-, OHB-, or EX537-treated samples. §, §§, and §§§§ represent a significant difference for *p* < 0.05, 0.01, or 0.0001, respectively, between H_2_O_2_-treated and untreated samples.

Regarding energy metabolism, the H_2_O_2_ addition to FA-corr lymphoblasts did not cause any significant effect (Figure 6), except for a slight decrease in the ATP/AMP ratio, which may depend on increased energy expenditure to counteract oxidative stress. Treatment with VPA, OHB, and EX527 concomitant with the oxidative insult also caused limited changes in FA-corr, in particular, an ATP/AMP ratio recovery, reaching levels similar to those of control cells not treated with H_2_O_2_. In addition, only OHB caused a coupled increase in OCR and ATP synthesis, probably due to the respiratory burst that this substrate induced on OxPhos activity.

In FA lymphoblasts, the H_2_O_2_ addition exacerbated the metabolic defects, further decreasing the electron transport between respiratory complexes I and III (Figure 6G) and the efficiency of OxPhos in the presence of both pyruvate plus malate (Figure 6A–C) and succinate (Figure 6D–F). Consequently, a reduction in cellular energy status (Figure 6H) and an increase in lipid accumulation and lipid peroxidation were observed. VPA treatment reversed all metabolic defects, causing a general recovery of OxPhos functions with an evident improvement in the coupling between OCR and ATP synthesis (Figure 6C,F), an increase in ALDH activity (Figure 6J) and the ATP/AMP ratio (Figure 6H), and a decrease in lipid content and oxidative state (Figure 6I and 6K, respectively). Conversely, OHB, due to its direct stimulating effect on the mitochondrion, induced further mitochondrial damage. In detail, OHB caused an increase in OCR but a decrease in ATP synthase, leading to further uncoupling (Figure 6A–F), associated with an increase in lipid accumulation (Figure 6I) and peroxidation (Figure 6K) and a reduction in cellular energy status (Figure 6H). EX527 exerted no effect on the H_2_O_2_-stressed FA lymphoblast metabolism. Similar results have been observed in primary fibroblasts (Figure S6).

## 4. Discussion

Cells affected by FA are characterized by a high level of oxidative stress [16,65,66] that is not counteracted by the antioxidant defenses [8,10], as confirmed by our data on the expression and the activity of CAT and GR, two of the main ROS-detoxifying enzymes. FA cells do not also improve their own antioxidant capacity following an oxidative stimulus, such as the hydrogen peroxide addition, suggesting their inability to trigger an adaptive response to oxidative stress. On the other hand, we have already shown how FA mononuclear cells are unable to express similar levels of antioxidant activity as control cells passing from the bone marrow hypoxic environment to the normoxic environment of the bloodstream [13].

It has been suggested that this inability may depend on the hypoacetylation of gene coding for antioxidant enzymes. Therefore, in this work, lymphoblasts and fibroblasts carrying the *FANC-A* gene mutation were treated with the following deacetylase inhibitors (HDACi): VPA, OHB, and EX527 [41,43,44]. The best results were obtained after treatment with VPA, which caused an improvement in the redox state and a partial recovery of aerobic energy metabolism function. In detail, VPA induced an enhancement of CAT and GR expression and activity after three hours from treatment, maintaining the result even 24 h after adding the drug. VPA treatment also improved the extent and efficiency of OxPhos thanks to the electron transport between respiratory complex I and III recovery. This effect could depend on oxidative stress accumulation reduction, as demonstrated by the MDA level decrement since the membrane phospholipids are one of the main targets of reactive oxygen species [67]. In addition, the OxPhos efficiency is also recovered due to the UCP2 expression reduction, as the electron transport chain function depends on the inner mitochondrial membrane integrity [68]. This aerobic metabolism enhancement causes, in turn, an increase in ATP availability, both because more is produced and because the cell must consume less to restore oxidative damage. A further target of VPA also appears to be the enzyme ALDH, which is co-involved in the detoxification of aldehydes, compounds that accumulate in FA cells and favor the production of oxidative stress and DNA damage. Because of the improvement in metabolic items, VPA-treated FA cells express lower levels of DRP1 than untreated cells because it is no longer necessary to promote mitochondrial fission to isolate damaged mitochondria for elimination [69]. The reduction in DRP1 levels results in the balance recovery between fusion and fission processes, which promote mitochondrial network formation, further improving OxPhos efficiency [70]. 

Furthermore, the restoration of mitochondrial function due to the VPA-induced improvement of the redox state could further alter gene expression by, again, playing on acetylation levels, as a ‘retrograde signaling’ exists between mitochondria and the nucleus in which the impairment of mitochondrial function causes changes in the expression of some nuclear genes [71]. For example, the gene expression appears regulated by some Krebs cycle metabolites [72], whose concentration can change depending on the electron transport chain functionality. Low citrate concentrations appear to induce DNA hypoacetylation, while increased levels of 2-hydroxyglutarate, the α-ketoglutarate antagonist, would cause its hypermethylation through a decreased NAD^+^/NADH ratio [73]. Thus, OxPhos function recovery could have changed the concentration of some metabolic intermediates upstream of cellular respiration, thereby modifying epigenetic signaling and the consequent gene expression. In other words, the positive loop between improved antioxidant defenses, the increased OxPhos efficiency, and mitochondrial network recovery triggered by VPA could, in turn, further modify gene expression, establishing a virtuous circle. Whatever the mechanisms by which VPA acts, at the cellular level, the result is a reduced MMC sensitivity in FA cells, suggesting less DBS accumulation. After all, Renaud et al. have already observed that another HDACi, trichostatin A, promoted DNA damage repair via homologous recombination (HR) instead of NHEJ [37]. However, it is possible to speculate that the positive effect on DNA repair depends not only on the type of mechanism used but also on a reduction in oxidative stress, aldehyde accumulation, and improved mitochondrial metabolism efficiency. In addition, to enhance the redox and energy status of FA cells by reducing DNA damage, VPA enhances the expansion and maintenance of hematopoietic progenitors by regulating the homeobox protein HOX-4B, a transcription factor involved in HSC self-renewal [47], suggesting a possible role in maintaining the hematopoietic stem cell pool and the aplasia risk reduction.

However, not all HDACi tested positively affected the redox balance and energy metabolism of FA cells. Treatment with OHB had opposite effects to those observed with VPA, although both molecules belong to HDACi classes I and II. In detail, OHB did not cause a decrease in oxidative damage, despite the slight increase in CAT and GR expression and the electron transfer between complexes I and III only after 24 h from the treatment. In contrast, OHB promoted further uncoupling of OxPhos and an accumulation of lipids and malondialdehyde. This opposite effect to VPA could be explained by the fact that OHB causes a respiratory burst that forces OxPhos activity, exacerbating the metabolic defect and increasing oxidative stress production. In addition, the unfunctional mitochondrial metabolism exacerbates the lipid accumulation in the cell, probably due to the increased AcetylCoA level [15], incrementing the risk of lipid peroxidation. These data suggest that metabolic and oxidative damages in FA cells depend on several factors, which should be corrected simultaneously to ensure a complete recovery of cell function. The opposite effects of VPA and OHB were observed even more after treatment with hydrogen peroxide, since whereas VPA induces, as in the case of basal conditions, an improvement in redox and biochemical parameters, OHB causes a further impairment of mitochondrial functions and an increase in oxidative stress.

EX527, a class III HDACi, does not display effects on FA cells, suggesting that, in FA cells, sirt1 does not play a pivotal role in aerobic metabolism regulation.

## 5. Conclusions

The data reported in this paper suggest that the *FANC-A* gene may be involved in determining DNA acetylation levels. Furthermore, VPA appears to have promising effects in improving the redox and metabolic aspect of FA cells, emphasizing, once again, that the accumulation of oxidative stress and altered energy metabolism play a strategic role in this pathology beyond the defect in DNA repair.

## Data Availability

All of the data is contained within the article and the Supplementary Materials.

## Supplementary Materials

The following supporting information can be downloaded at https://www.mdpi.com/article/10.3390/antiox12051100/s1. Figure S1: Comparison of biochemical activities in the healthy donor-derived and FA-corr lymphoblast cell lines; Figure S2: Comparison of biochemical activities in the healthy donor-derived and FA-corr primary fibroblasts; Figure S3: Modulation of Catalase and Glutathione Reductase expression and activity in FA fibroblasts treated with VPA, OHB, or EX527; Figure S4: VPA, OHB, and EX527 effects on energy metabolism parameters and lipid peroxidation in FA fibroblasts; Figure S5: Modulation of Catalase and Glutathione Reductase expression and activity in FA fibroblasts treated with VPA, OHB, or EX527 after the hydrogen peroxide addition; Figure S6: VPA, OHB, and EX527 effects on energy metabolism parameters and lipid peroxidation in FA fibroblasts after the hydro-gen peroxide addition.
