# Supplementary material for: Effects of Deacetylase Inhibition on the Activation of the Antioxidant Response and Aerobic Metabolism in Cellular Models of Fanconi Anemia
Source: Antioxidants (Basel). 2023 May 15;12(5):1100. doi: 10.3390/antiox12051100 (PMC10215951; doi:10.3390/antiox12051100)
Supplement: Supplementary file 1 [file antioxidants-12-01100-s001.zip › antioxidants-2249440-supplementary.pdf]

Figure S1

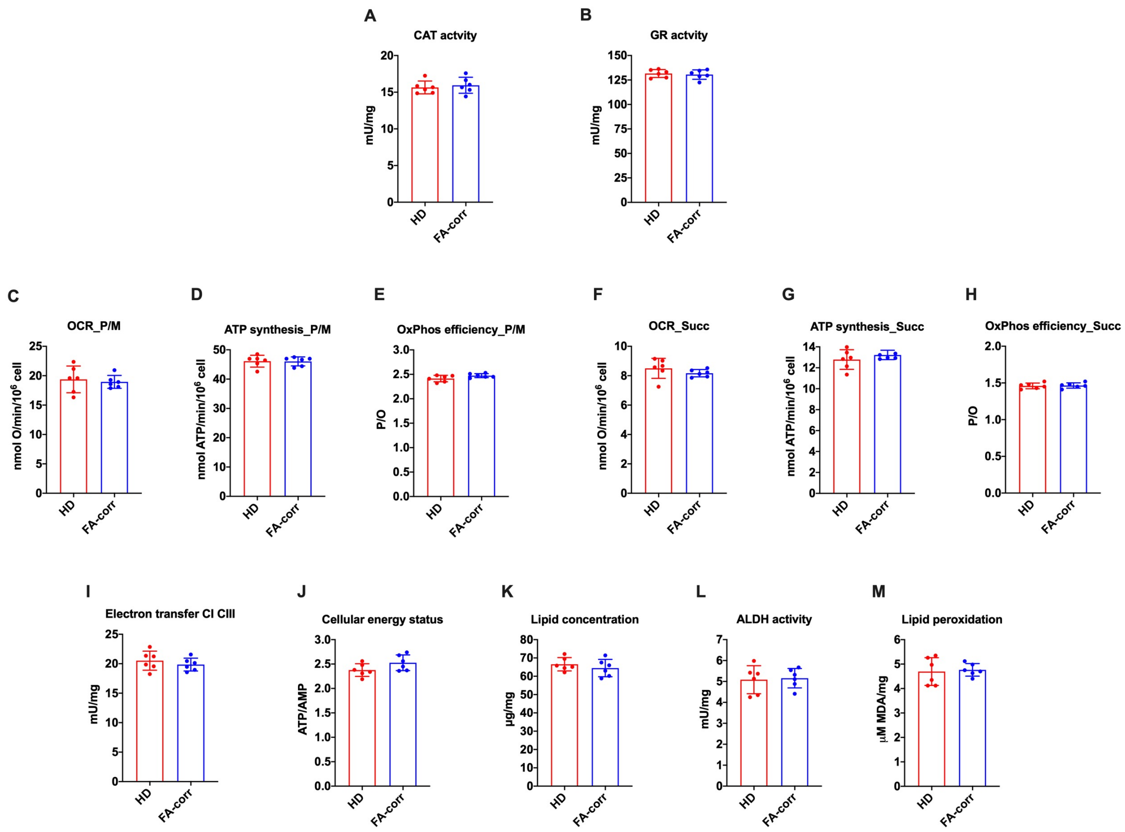

Figure S1: Comparison of biochemical activities in the healthy donor-derived and FA-corr lymphoblast cell lines. The following biochemical activities have been tested in the healthy donor (HD) and FA-corr lymphoblast cell lines. (A) Catalase (CAT) activity; (B) Glutathione reductase (GR) activity; (C) Oxygen Consumption Rate (OCR) in the presence of pyruvate plus malate (P/M); (D) ATP synthesis through F_oF_1 ATP synthase in the presence of P/M; (E) P/O ratio as an OxPhos efficiency marker in the presence of P/M; (F) Oxygen Consumption Rate (OCR) in the presence of succinate (Succ); (G) ATP synthesis through F_oF_1 ATP synthase in the presence of Succ; (H) P/O ratio as an OxPhos efficiency marker in the presence of Succ; (I) Electron transfer between complexes I and III; (J)ATP/AMP ratio as a cellular energy status marker; (K) Cellular lipid concentration; (L) Aldehyde dehydrogenase (ALDH) activity; (M) Malondialdhyede (MDA) intracellular concentration, as a lipid peroxidation marker. Data are reported as mean ± SD, and each graph is representative of 6 independent experiments. Statistical significance was tested with a one-way ANOVA, and no significant differences have been observed.

Figure S2

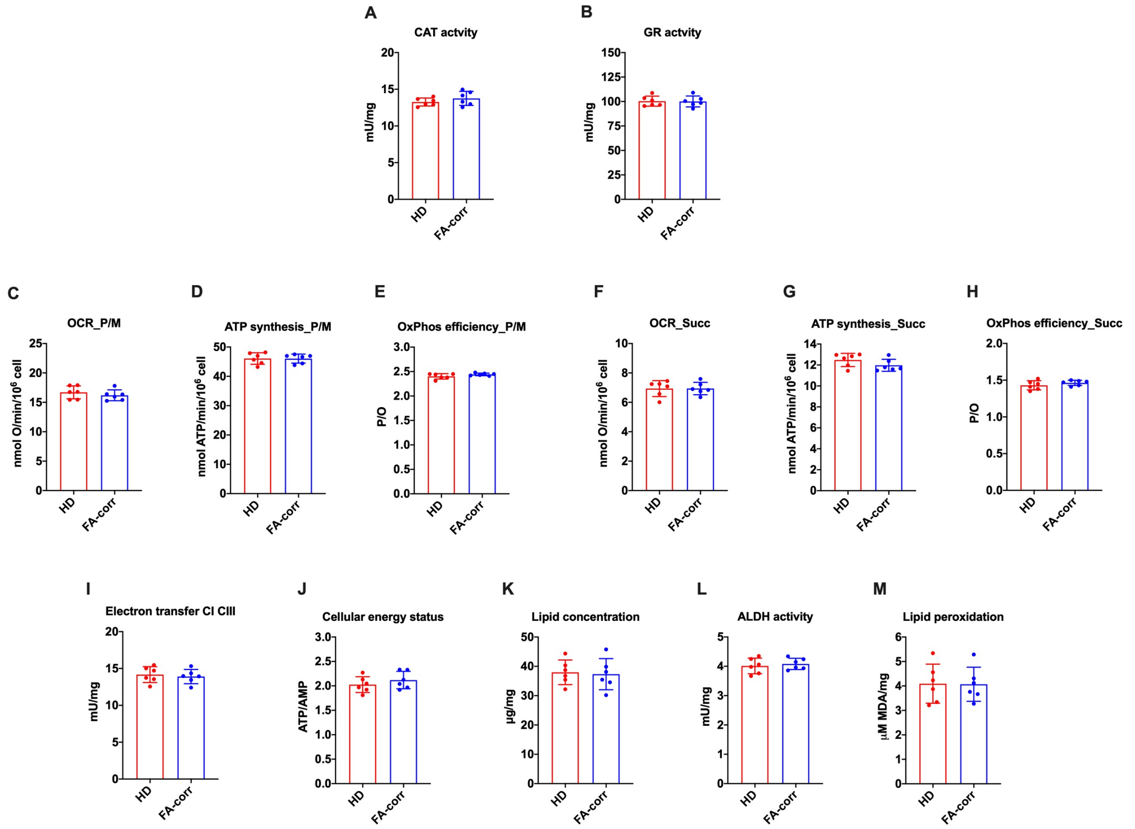

Figure S2: Comparison of biochemical activities in the healthy donor-derived and FA-corr primary fibroblasts. The following biochemical activities have been tested in the healthy donor (HD) and FA-corr primary fibroblasts. (A) Catalase (CAT) activity; (B) Glutathione reductase (GR) activity; (C) Oxygen Consumption Rate (OCR) in the presence of pyruvate plus malate (P/M); (D) ATP synthesis through F_oF_1 ATP synthase in the presence of P/M; (E) P/O ratio as an OxPhos efficiency marker in the presence of P/M; (F) Oxygen Consumption Rate (OCR) in the presence of succinate (Succ); (G) ATP synthesis through F_oF_1 ATP synthase in the presence of Succ; (H) P/O ratio as an OxPhos efficiency marker in the presence of Succ; (I) Electron transfer between complexes I and III; (J)ATP/AMP ratio as a cellular energy status marker; (K) Cellular lipid concentration; (L) Aldehyde dehydrogenase (ALDH) activity; (M) Malondialdhyede (MDA) intracellular concentration, as a lipid peroxidation marker. Data are reported as mean ± SD, and each graph is representative of 6 independent experiments. Statistical significance was tested with a one-way ANOVA, and no significant differences have been observed.

Figure S3

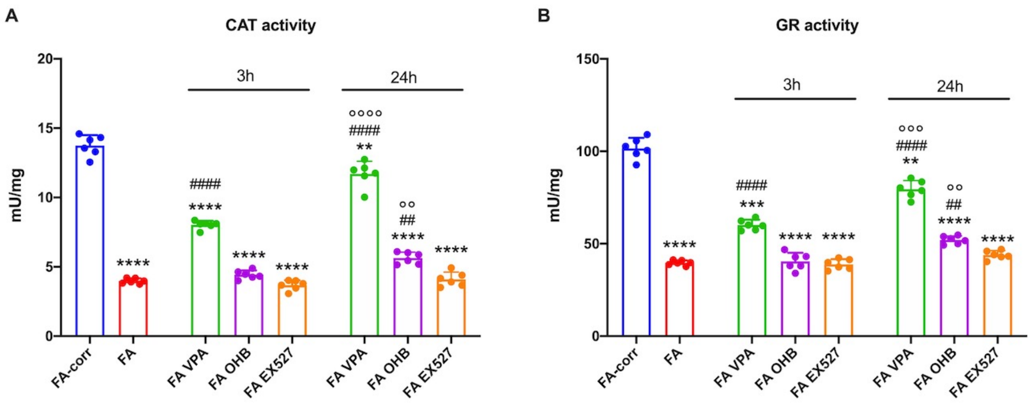

Figure S3: Modulation of Catalase and Glutathione Reductase expression and activity in FA fibroblasts treated with VPA, OHB, or EX527.

(A) Catalase (CAT) activity; (B) Glutathione reductase (GR) activity. In each panel, the effects were evaluated in fibroblasts after 3 and 24 hours from VPA, OHB, or EX527 addition. Data are reported as mean ± SD, and each graph is representative of 6 independent experiments. Statistical significance was tested with a one-way ANOVA. **, *** and **** represent a significant difference for p<0.01, 0.001, or 0.0001, respectively, between FA and FA-corr cells used as control. ## and #### represent a significant difference for p<0.01 or 0.0001, respectively, between untreated and treated FA cells. °°, °°°, and °°°° represent a significant difference for p<0.01, 0.001, and 0.0001 between the same treatment at 3 and 24 hours.

Figure S4

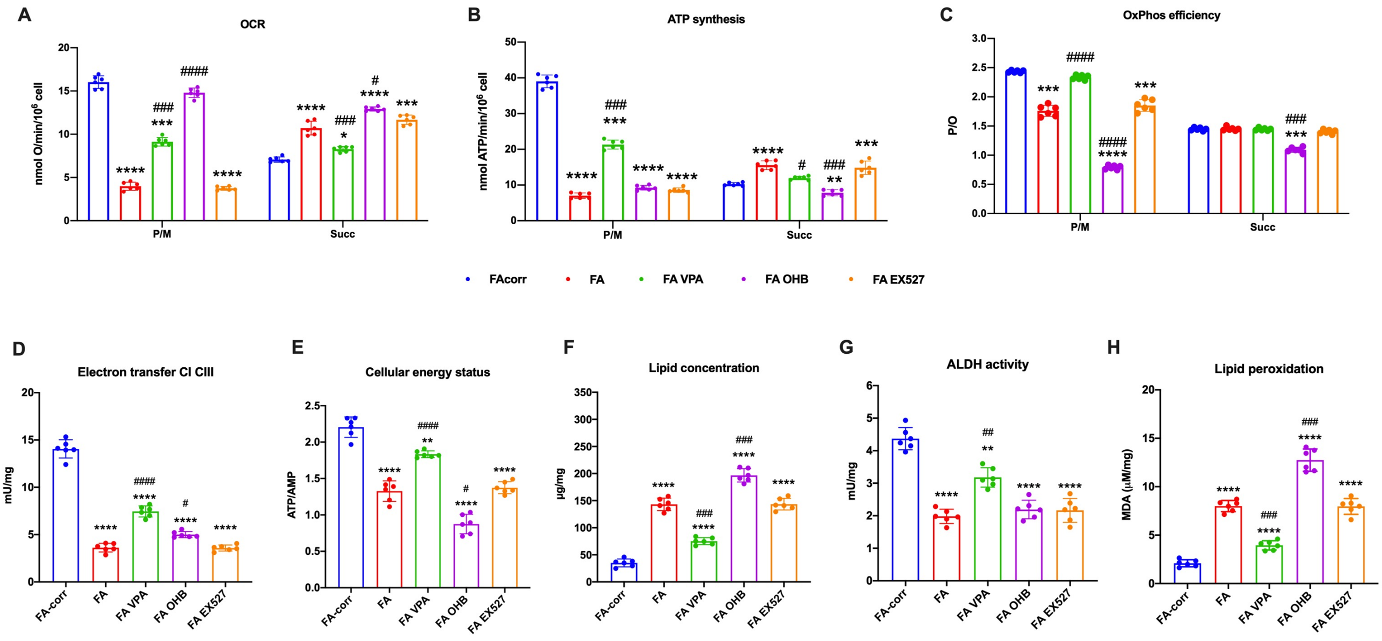

Figure S4: VPA, OHB, and EX527 effects on energy metabolism parameters and lipid peroxidation in FA fibroblasts
(A) Oxygen Consumption Rate (OCR); (B) ATP synthesis through F_oF_1 ATP synthase; (C) P/O ratio as an OxPhos efficiency marker. For panels A-C, pyruvate/malate (P/M) and succinate (Succ) have been employed as respiratory substrates. (D) Electron transfer between complexes I and III; (E) ATP/AMP ratio as a cellular energy status marker; (F) Cellular lipid concentration; (G) Aldehyde dehydrogenase (ALDH) activity; (H) Malondialdhyede (MDA) intracellular concentration, as a lipid peroxidation marker. In each panel, the effects were evaluated in fibroblasts after 24 hours from VPA, OHB, or EX527 addition. Data are reported as mean ± SD, and each graph is representative of 6 independent experiments. Statistical significance was tested opportunely with a one-way ANOVA or two-way ANOVA. *, **, ***, and **** represent a significant difference for p<0.05, 0.01, 0.001, or 0.0001, respectively, between FA and FA-corr cells used as control. #, ##, ###, and #### represent a significant difference for p<0.05, 0.01, 0.001, or 0.0001, respectively, between untreated and treated FA cells.

Figure S5

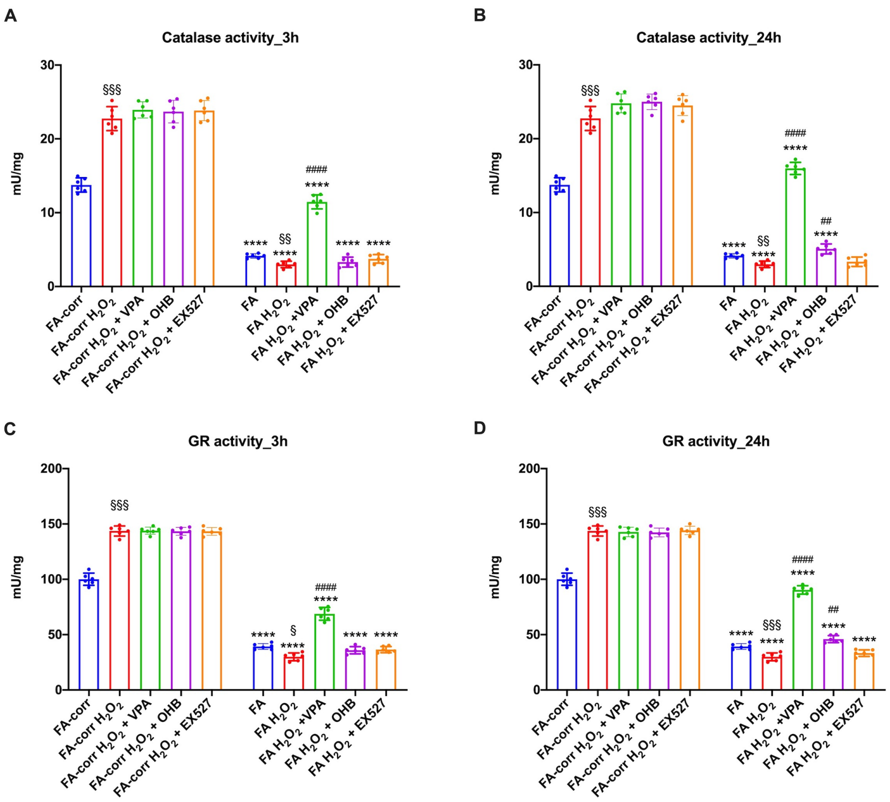

Figure S5: Modulation of Catalase and Glutathione Reductase expression and activity in FA fibroblasts treated with VPA, OHB, or EX527 after the hydrogen peroxide addition.

In each graph, 0.5 mM hydrogen peroxide was added to induce oxidative insult. (A) and (B) Catalase (CAT) activity after 3h and 24h from VPA, OHB, or EX527 addition, respectively; (C) and (D) Glutathione reductase (GR) activity after 3h and 24h from VPA, OHB, or EX527 addition, respectively. Data are reported as mean ± SD, and each graph is representative of 6 independent experiments. Statistical significance was tested with a one-way ANOVA. **** represents a significant difference for p 0.0001 between FA and FA-corr cells in the same treatment condition. ## and #### represent a significant difference for $p<0.01$ or 0.0001, respectively, between untreated and VPA-, OHB-, or EX537-treated samples. §, §§, and §§§ represents a significant difference for $p<0.05$, 0.01, or 0.001, respectively, between H_2O_2-treated and untreated samples.

Figure S6

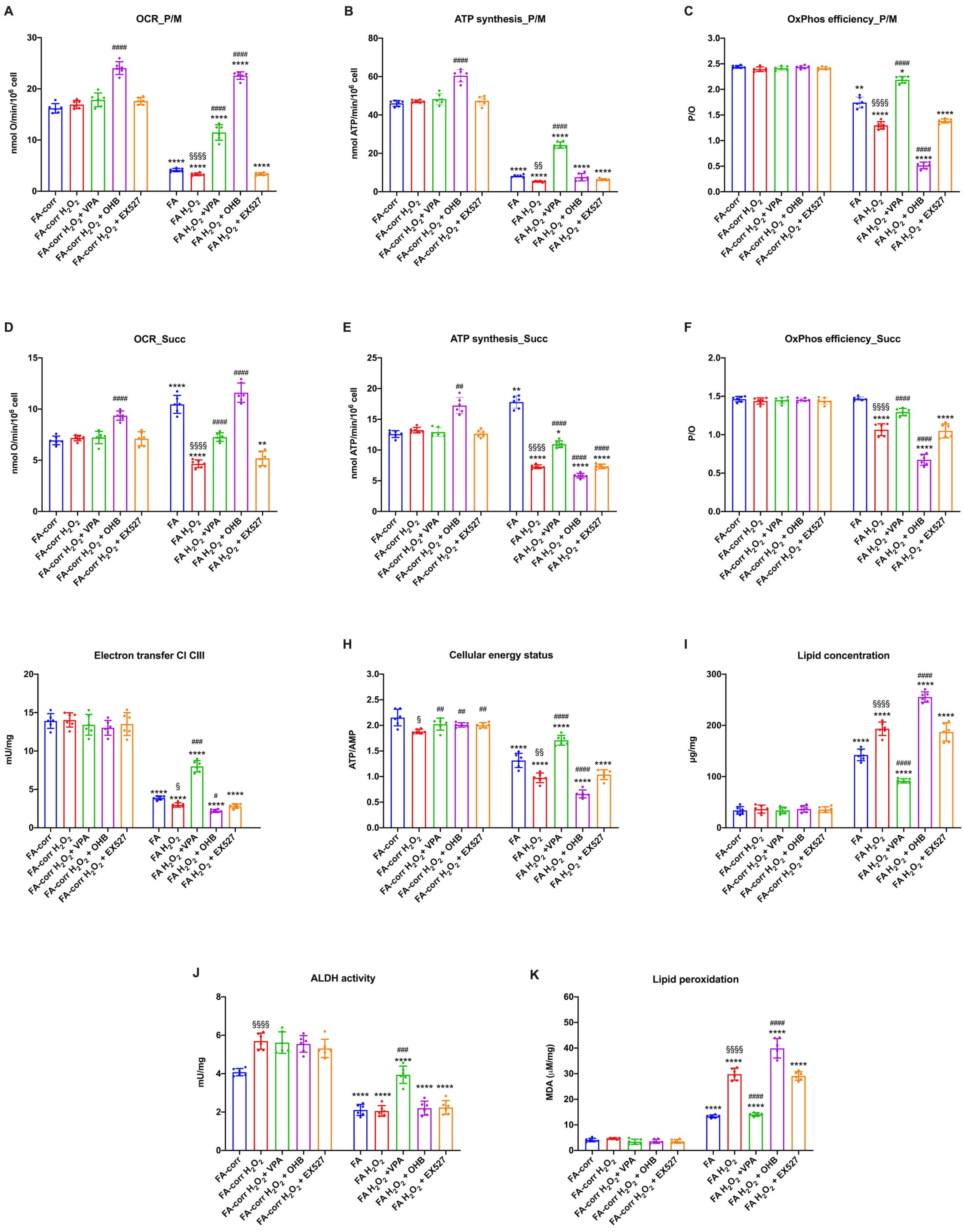
A
OCR_P/M
nmol O/min/10⁶ cell
B
ATP synthesis_P/M
nmol ATP/min/10⁶ cell
C
OxPhos efficiency_P/M
P/O
D
OCR_Succ
nmol O/min/10⁶ cell
E
ATP synthesis_Succ
nmol ATP/min/10⁶ cell
F
OxPhos efficiency_Succ
P/O
G
Electron transfer CI CIII
mU/mg
H
Cellular energy status
ATP/AMP
I
Lipid concentration
µg/mg
J
ALDH activity
mU/mg
K
Lipid peroxidation
MDA (µM/mg)
FA-corr
FA-corr H2O2
FA-corr H2O2 + VPA
FA-corr H2O2 + OHB
FA-corr H2O2 + EX527
FA
FA H2O2
FA H2O2 +VPA
FA H2O2 + OHB
FA H2O2 + EX527

Figure S6: VPA, OHB, and EX527 effects on energy metabolism parameters and lipid peroxidation in FA fibroblasts after the hydrogen peroxide addition.
(A) Oxygen Consumption Rate (OCR) in the presence of pyruvate plus malate (P/M); (B) ATP synthesis through F_oF_1 ATP synthase in the presence of P/M; (C) P/O ratio in the presence of P/M as an OxPhos efficiency marker; (D) Oxygen Consumption Rate (OCR) in the presence of succinate (Succ); (E) ATP synthesis through F_oF_1 ATP synthase in the presence of Succ; (F) P/O ratio in the presence of Succ as an OxPhos efficiency marker; (G) Electron transfer between complexes I and III; (H) ATP/AMP ratio as a cellular energy status marker; (I) Cellular lipid concentration; (J) Aldehyde dehydrogenase (ALDH) activity; (K) Malondialdehyde (MDA) intracellular concentration, as a lipid peroxidation marker. In each panel, the effects were evaluated in fibroblasts after 24 hours from VPA, OHB, or EX527 addition. Data are reported as mean ± SD, and each graph is representative of 6 independent experiments. Statistical significance was tested opportunely with a one-way ANOVA or two-way ANOVA. *, **, and **** represent a significant difference for p<0.05, 0.01, or 0.0001, respectively, between FA and FA-corr cells in the same treatment condition. #, ##, ###, and #### represent a significant difference for p<0.05, 0.01, 0.001, or 0.0001, respectively, between untreated and VPA-, OHB-, or EX537-treated samples. §, §§, and §§§§ represents a significant difference for p<0.05, 0.01, or 0.0001, respectively, between H2O2-treated and untreated samples.
